# Single and joint impact of type 2 diabetes and of congestive heart failure on albuminuria: Data from subgroup analysis and data on moderate albuminuria

**DOI:** 10.1016/j.dib.2022.107817

**Published:** 2022-01-10

**Authors:** Christoph H. Saely, Maximilian Maechler, Alexander Vonbank, Lukas Sprenger, Arthur Mader, Barbara Larcher, Daniela Zanolin-Purin, Andreas Leiherer, Axel Muendlein, Heinz Drexel

**Affiliations:** a Vorarlberg Institute for Vascular Investigation and Treatment (VIVIT), Carinagasse 47, Feldkirch AT-6800, Austria; b Private University in the Principality of Liechtenstein, Dorfstrasse 24, Triesen FL-9495, Liechtenstein; c Department of Medicine I, Academic Teaching Hospital Feldkirch, Carinagasse 47, Feldkirch AT-6800, Austria; d Drexel University College of Medicine, 2900 W Queen Ln, Philadelphia, PA 19129, United States

**Keywords:** Albumin–creatinine ratio, Heart failure, Diabetes mellitus, Gender differences, Moderate albuminuria

## Abstract

We investigated 180 consecutive patients with congestive heart failure (CHF), of whom 83 had type 2 diabetes (T2DM) and 97 did not have diabetes as well as 223 controls without CHF, of whom 39 had T2DM and 184 did not have diabetes. Data was recorded by standardized interviews and by standardized examination protocols at our institution and were extracted from medical records. Here, we analyzed data on gender differences. Further, we examined the effect of CHF and T2DM on moderate albuminuria, i.e. on an albumin-creatinine ratio (ACR) of 30–300 mg/g. Table 1 shows baseline characteristics of our patients stratified by gender. Table 2 gives ACRs and prevalence rates of albuminuria separately for men and women.

In logistic regression analyses adjusting for age, sex, body mass index, LDL cholesterol, history of smoking, history of hypertension, use of statins, ACE inhibitors/angiotensin II receptor blockers, aldosterone antagonists and other antihypertensive medication CHF and T2DM predicted the prevalence of albuminuria in a mutually independent manner in men (OR 4.93 [95% CI 1.76–13.85]; *p* = 0.002 and OR 2.38 [1.11–5.11]; *p* = 0.027, respectively), as well as in women (OR 5.66 [95% CI 1.76–18.20]; *p* = 0.004 and OR 3.53 [1.38–9.08]; *p* = 0.009, respectively). There was no significant interaction between gender and CHF or T2DM regarding the presence of albuminuria (*p* = 0.933 and 0.533, respectively), indicating that the association of CHF and T2DM with albuminuria did not differ significantly between men and women.

In multivariate analysis of covariance, CHF and T2DM proved to be independent predictors of ACR in women after adjustment for age, sex, body mass index, LDL cholesterol, history of smoking, history of hypertension, use of statins, ACE inhibitors/angiotensin II receptor blockers, aldosterone antagonists and other antihypertensive medication (*F* = 5.38; *p* = 0.022 and *F* = 4.95; *p* = 0.028, respectively); for men the corresponding *F*-values were 2.70; *p* = 0.102 and 3.12; *p* = 0.079, respectively. There was no significant interaction between gender and CHF or T2DM regarding ACR (*p* = 0.464 and 0.202, respectively), indicating that the association of CHF and T2DM with the ACR did not differ significantly between men and women.

Regarding moderate albuminuria, both CHF and T2DM predicted moderate albuminuria adjusted in a mutually independent manner after the adjustments described above, with ORs of 4.75 [95% CI 2.16–10.45]; p< 0.001 and OR 2.08 [1.13–3.83]; p=0.018, respectively.

The data set presented here could be reused with similar patient cohorts for pooled analysis.

## Specifications Table

**Table:** 

Subject	Cardiology and Cardiovascular Medicine
Specific subject area	Associations of congestive heart failure and type 2 diabetes with albuminuria, taking into account potential gender differences.
Type of data	Table Dataset
How the data were acquired	Consecutive patients admitted for congestive heart failure (CHF) to a tertiary care center were enrolled; as controls we used patients without signs and symptoms of congestive heart failure in whom coronary artery disease was ruled out angiographically. Information on conventional cardiovascular risk factors were obtained by standardized interviews. Biochemical measurements were obtained from fasting venous blood or urine samples, taken within one day of enrolment. Systolic and diastolic blood pressure was measured by the Riva-Rocci method under resting conditions in a sitting position at the day of inclusion and at least five hours after hospitalization. Height and weight were recorded at the day of admission, and body mass index was calculated as body weight (kg) / height (m)^2^. Left ventricular ejection fraction was obtained by transthoracic echocardiography. Data was organized in an encrypted dataset using IBM SPSS Statistics 24.0.0.0 for Windows (SPSS, Inc., USA).
Data format	Analyzed Filtered Raw
Description of data collection	Diagnosis of congestive heart failure was made according to the 2016 European Society of Cardiology (ESC) definition [2]. As controls we used 223 consecutive patients who had no signs or symptoms of CHF and in whom significant coronary artery disease (CAD) was ruled out angiographically. Significant CAD was defined as at least one lesion with a stenosis of 50% or more on coronary angiogram, as we already described before [3]. All patients were routinely screened for diabetes using HbA1c and fasting plasma glucose if a diagnosis of diabetes had not been already established previously. The diagnosis of type 2 diabetes (T2DM) was made according to 2020 ADA criteria [4]. Patients with acute coronary syndrome (ACS) and/or patients with type 1 diabetes (C-peptide negative) were not enrolled.
Data source location	• Institution: Vorarlberg Institute for Vascular Investigation and Treatment (VIVIT) and Department of Medicine I, Academic Teaching Hospital Feldkirch • City/Town/Region: Feldkirch, Vorarlberg • Country: Austria
Data accessibility	Repository name: Mendeley Data Data identification number: 10.17632/7cp325hk29.1 Direct URL to data: https://data.mendeley.com/datasets/7cp325hk29/1 data was fully anonymised and therefore no access control is needed
Related research article	Saely CH, Maechler M, Vonbank A, Sprenger L, Mader A, Larcher B, Zanolin-Purin D, Leiherer A, Muendlein A, Drexel H. Single and joint impact of type 2 diabetes and of congestive heart failure on albuminuria. J Diabetes Complications. 2021 Dec;35(12):108046. doi: 10.1016/j.jdiacomp.2021.108046. Epub 2021 Sep 12. PMID: 34598838.

## Value of the Data

• T2DM and congestive heart failure are highly prevalent and often are combined in one patient. Albuminuria predicts cardiovascular morbidity and mortality in T2DM [5,6], as well as jn CHF [7,8]; the prevalence of albuminuria is increased both in T2DM [9] and CHF [5], but the single and joint effects of T2DM on albuminuria in gender specific analyses had not yet been addressed.
• The data benefit researchers as well as health care professionals in the fields of diabetology, cardiology, nephrology and general internal medicine.
• Our data should stimulate the development of study protocols to gain further insight into the interplay between albuminuria, congestive heart failure and T2DM, including prospective investigations and interventional studies.

## 1 Data Description

• Table 1 Shows the baseline characteristics in men and women of our study population with regard to the presence of both CHF and T2DM [1]. The values listed represent the mean and the standard deviation with a confidence interval of 95% unless denoted otherwise. Statistical significance was defined as two-tailed *p*-value of 0.05.

**Table 1:** **Baseline characteristics of study population**, mean ± SD (95% CI), statistical significance was defined as two-tailed *p* value of 0.05.

	**CHF -**			**CHF +**		
	**T2DM-***n* = 184 (45.7%)	**T2DM+***n* = 39 (9.7%)	***p*-value**	**T2DM-***n* = 97 (24.1%)	**T2DM+***n* = 83 (20.6%)	***p*-value**
Age (years)						
Men	57 ± 11 (54.4–58.7)	64 ± 11 (59.3–68.7)	0.006	69 ± 15 (64.9–72.6)	72 ± 11 (68.6–75.0)	0.364
Women	62 ± 10 (60.1–64.3)	63 ± 7 (59.8–67.2)	0.618	78 ± 16 (72.8–83.0)	76 ± 13 (71.6–80.8)	0.399
BMI (kg/m^2^)						
Men	28 ± 4 (26.8–28.3)	30 ± 3 (28.2–30.9)	0.004	26 ± 5 (25.1–27.7)	30 ± 6 (28.1–31.2)	0.001
Women	28 ± 5 (26.6–28.7)	34 ± 6 (31.0–36.8)	<0.001	27 ± 6 (25.0–28.6)	28 ± 9 (24.8–31.0)	0.959
History of Smoking (%)						
Men	68.7	90.9	**0.034**	69.0	68.6	0.970
Women	41.2	29.4	0.364	35.9	34.4	0.894
History of hypertension (%)						
Men	57.6	81.8	**0.034**	62.6	82.4	**0.019**
Women	55.3	88.2	**0.011**	71.8	87.5	0.107
LDL-C (mg/dl)						
Men	132 ± 35 (125–139)	129 ± 37 (112–145)	0.778	121 ± 54 (107–136)	129 ± 68 (109–148)	0.516
Women	143 ± 38 (135–151)	110 ± 38 (90–129)	**0.004**	125 ± 43 (110–139)	135 ± 53 (114–155)	0.359
Use of antihypertensive drugs (%)						
Men	65.7	81.8	0.140	100.0	100.0	1.000
Women	55.3	88.2	**0.011**	97.4	100.0	0.362
Use of ACEi or ARBs (%)						
Men	32.3	36.4	0.716	73.2	64.0	0.306
Women	29.4	41.2	0.340	47.4	59.4	0.316
Use of aldosterone antagonists (%)						
Men	1.0	0.0	0.636	44.6	36.0	0.366
Women	2.4	5.9	0.432	23.1	9.4	0.125
Prevalence of albuminuria (%) *						
Men	9.1	22.7	0.070	36.2	56.9	**0.031**
Women	8.2	23.5	0.063	41.0	71.9	**0.009**
Prevalence of moderately increased albuminuria (%) °						
Men	7.2	19.0	0.091	33.9	47.6	0.171
Women	8.2	23.5	0.063	36.1	65.4	**0.023**

**CHF** = congestive heart failure, **T2DM** = type 2 diabetes mellitus, **BMI** = body mass index, **LDL-C** = low-density lipoprotein cholesterol, **ACEi** = ACE Inhibitor, **ARB** = Angiotensin receptor blocker.

⁎ defined as ACR ≥ 30 mg/g defined as ACR 30–300 mg/g.
• Table 2 Shows ACRs and the prevalence of albuminuria in men and women with regard to the presence of both CHF and T2DM. The values in the table are listed in the same way as described for Table 1.

**Table 2:** **ACR and prevalence of albuminuria in men and women,** median [interquartile range], statistical significance was defined as two-tailed *p* value of 0.05.

	**CHF -**			**CHF +**		
	**T2DM-***n* = 99 (43.0%)	**T2DM+***n* = 22 (9.6%)	***p*-value**	**T2DM-***n* = 58 (25.2%)	**T2DM+***n* = 51 (22.2%)	***p*-value**
ACR (mg/g)						
Men	7.8 [4.8–15.1]	13.1 [8.4–29.9]	**0.003**	20.0 [8.5–73.6]	59.0 [16.0–168.0]	**0.006**
Women	11.9 [6.6–17.7]	16.7 [6.2–31.5]	0.188	25.0 [9.0–82.0]	73.5 [16.0–222.8]	**0.033**
Prevalence of albuminuria (%) *						
Men	9.1	22.7	0.070	36.2	56.9	**0.031**
Women	8.2	23.5	0.063	38.2	62.7	**0.009**

**CHF** = congestive heart failure, **T2DM** = type 2 diabetes mellitus, **ACR** = albumin–creatinine ratio.

⁎ defined as ACR ≥ 30 mg/g.
• Dataset: Features the data of our study population including the presence of CHF and T2DM, gender, ACR and prevalence of albuminuria. The data are pseudonymized as each participant was assigned to consecutive numbers (“ID”) on the basis of date of inclusion. The variables “Diabetes_mellitus_type_2” and “congestive_heart_failure” distinguish if subjects are suffering from T2DM and/or CHF, whereas “0” encodes “no” and “1” stands for “yes”. “Sex” describes the biological sex of subjects, whereas “0” stands for “female” and “1” for “male” patients. The variable “albumine_creatinine_ratio” describes the quantity of albumin-creatinine ratio (ACR) in mg/g. Finally, the variable “moderate_albuminuria” states if subjects suffer from moderate albuminuria, which is defined as an ACR of 30–300 mg/g, and as before “0” stands for “no” and “1” for yes.
• Questionnaire: The questionnaire used for the standardized interview and examination of every subject.

## 2 Experimental Design, Materials and Methods

We ruled in consecutive patients who were admitted to the LKH Feldkirch, a tertiary care center in Austria, for congestive heart failure. Eligible patients were identified via the hospital record system. As a control group we used consecutive patients who were admitted for coronary angiography with no signs or symptoms of CHF according to the 2016 European Society of Cardiology (ESC) definition [2] and in whom coronary artery disease was ruled out by angiography. Demographics, patient history and relevant medical findings were obtained by a standardized interview and examination. Biochemical measurements were obtained from fasting venous blood or urine samples, taken within one day of inclusion.

The diagnosis of type 2 diabetes (T2DM) was made according to 2020 ADA criteria [4]. Diagnosis of congestive heart failure was made according to the 2016 European Society of Cardiology (ESC) definition [2].

ACR was measured on either cobas c501/c502, c702 or Integra 800 (Roche, Switzerland) using fasting morning urine (ACN 253 or ACN 8253 for albumin by immunoturbidimetry and ACN 691 or ACN 8691 for creatinine by Jaffe reaction). Moderate albuminuria was defined as a range of ACR of 30 mg/g or more and 300 mg/g or less.

NT-proBNP was measured on cobas e601, e602, e801 or Elecsys 2010 (Roche, Switzerland) using corresponding, commercially available, immunological tests. Hba1c-levels were measured on Adams HA-8160 (Arkray, Japan) using High Performance Liquid Chromatography.

Between-group differences were tested for statistical significance using Mann–Whitney–U test for continuous variables and by applying the chi-squared test for categorical variables. Analyses of covariance (ANCOVA) was performed using the general linear model approach, and logistic regression analyses were applied. Results are described as mean median [interquartile range] if not denoted otherwise. Statistical significance was defined as two-tailed *p* value of 0.05. All statistical analyses were performed with the software package IBM SPSS Statistics 24.0.0.0 for Windows (SPSS, Inc., USA).

## Ethics Statements

We hereby state that the research has been carried out in accordance with The Code of Ethics of the World Medical Association (Declaration of Helsinki), that the Ethics Committee of the University of Innsbruck approved this study (EK-2-2008/0017) and that all participants gave written informed consent.

## CRediT authorship contribution statement

**Christoph H. Saely:** Conceptualization, Methodology, Validation, Writing – review & editing, Supervision. **Maximilian Maechler:** Validation, Formal analysis, Investigation, Writing – original draft, Visualization. **Alexander Vonbank:** Writing – review & editing. **Lukas Sprenger:** Investigation, Visualization. **Arthur Mader:** Investigation, Visualization. **Barbara Larcher:** Investigation, Visualization. **Daniela Zanolin-Purin:** Investigation, Formal analysis. **Andreas Leiherer:** Writing – review & editing. **Axel Muendlein:** Writing – review & editing. **Heinz Drexel:** Conceptualization, Supervision, Project administration.

## Declaration of Competing Interest

The authors declare that they have no known competing financial interests or personal relationships that could have appeared to influence the work reported in this paper.

## Appendix Supplementary materials

Image, application 1
