# Supplementary material for: Single and joint impact of type 2 diabetes and of congestive heart failure on albuminuria: Data from subgroup analysis and data on moderate albuminuria
Source: Data Brief. 2022 Jan 10;40:107817. doi: 10.1016/j.dib.2022.107817 (PMC8762350; doi:10.1016/j.dib.2022.107817)
Supplement: Supplementary file 1 [file mmc1.doc]

**BASELINE**

**PatID: ________**

| **Date:** |
| --- |
| **Name:**  **Date of birth:**  **Sex:** |

| **Reason for admission (text)** | | | | | | | |  |
| --- | --- | --- | --- | --- | --- | --- | --- | --- |
|  | | | **No** | | **Yes** | | **Comments** |  |
| **ACS** | | |  | |  | |  |  |
| **signs of infection at time of admission** | | |  | |  | |  |  |
| **known diagnosis of heart failure before time of admission** | | |  | |  | |  |  |
| **acute HF** | | |  | |  | |  |  |
| **ischaemic HF** | | |  | |  | |  |  |
| **non-ischaemic HF** | | |  | |  | |  |  |
| **NYHA** | | | **I / II / III / IV** | | | | |  |
| **first hospitalization because of HF** | | |  | |  | |  |  |
| **time since last hospitalization because of HF** | | | (months) | | | | |  |
|  | | | | | | | |  |
| **atrial fibrillation** | | |  | |  | |  |  |
| **type of AF** | | | **first diagnosis**  **paroxysmal**  **persistent AF**  **permanent AF** | | | | |  |
|  | | |  | | | | |  |
| **known DM:**  Yes  No Type I  Type II   first diagnoses:………………….(duration in years)  insuline:  No  Yes, since.... hypoglycaemic events / month: severe hypoglycaemic events / last year (need of assistance): | | | | | | | | |
|  | | | | | | | | |
|  | No | Yes | | Date | | Comments | | |
| CAD |  |  | |  | |  | | |
| insult |  |  | |  | |  | | |
| PAD |  |  | |  | |  | | |
| valve surgery |  |  | |  | |  | | |
| other arrhythmias than AF |  |  | |  | |  | | |
| cardiac devices |  |  | |  | |  | | |
| hypertension |  |  | |  | |  | | |
| COPD |  |  | |  | |  | | |
|  |  |  | |  | |  | | |

| Admission | |
| --- | --- |
| **Time of blood sample** |  |
| height (cm) |  |
| weight (kg) |  |
